# A model of microsaccade-related neural responses induced by short-term depression in thalamocortical synapses

**DOI:** 10.3389/fncom.2013.00047

**Published:** 2013-04-23

**Authors:** Wu-Jie Yuan, Olaf Dimigen, Werner Sommer, Changsong Zhou

**Affiliations:** ^1^Department of Physics, Institute of Computational and Theoretical Studies, Centre for Non-linear Studies and the Beijing-Hong Kong-Singapore Joint Centre for Nonlinear and Complex Systems (Hong Kong), Hong Kong Baptist UniversityKowloon Tong, Hong Kong, China; ^2^College of Physics and Electronic Information, Huaibei Normal UniversityHuaibei, China; ^3^Department of Psychology, Humboldt University at BerlinBerlin, Germany

**Keywords:** short-term depression, microsaccades, feedforward network, visual fading, fixation

## Abstract

Microsaccades during fixation have been suggested to counteract visual fading. Recent experiments have also observed microsaccade-related neural responses from cellular record, scalp electroencephalogram (EEG), and functional magnetic resonance imaging (fMRI). The underlying mechanism, however, is not yet understood and highly debated. It has been proposed that the neural activity of primary visual cortex (V1) is a crucial component for counteracting visual adaptation. In this paper, we use computational modeling to investigate how short-term depression (STD) in thalamocortical synapses might affect the neural responses of V1 in the presence of microsaccades. Our model not only gives a possible synaptic explanation for microsaccades in counteracting visual fading, but also reproduces several features in experimental findings. These modeling results suggest that STD in thalamocortical synapses plays an important role in microsaccade-related neural responses and the model may be useful for further investigation of behavioral properties and functional roles of microsaccades.

## 1. Introduction

When the eyes fixate at a stationary object, they are never completely motionless, but perform involuntary, very small eye movements. These fixational eye movements are composed of three different types of movement: tremor, microsaccades, and drift. Tremor is an aperiodic, high-frequency fixational eye movement with the smallest amplitude of these three types of fixational eye movements. Microsaccades are involuntary jerk-like fixational eye movements. Drift is a typical fixational eye movement taking place between microsaccades with the slowest velocity of all the three types. Microsaccades are the largest and fastest fixational eye movements. It has been experimentally observed that microsaccades cause more variability in neuronal responses than both tremor and drift (Gur et al., 1997; Martinez-Conde, 2006). The most prominent contribution to fixational eye movements is generated by microsaccades (Rolfs, 2009). Therefore, both experimental and theoretical works have mainly focused on the role of microsaccades during fixation.

Over the past decade, the behavioral properties and functional roles of microsaccades have been widely investigated (for reviews, see Martinez-Conde et al., 2004, 2009; Rolfs, 2009). Importantly, it was found that the visual world quickly fades from view in the absence of fixational eye movements (Ditchburn and Ginsborg, 1952; Riggs and Ratliff, 1952). This suggests that microsaccades play an important functional role in counteracting visual fading during fixation (Ditchburn and Ginsborg, 1952; Martinez-Conde, 2006). Recently, the mechanism of microsaccades for counteracting perceptual fading has received much research interest. Several studies have assumed that microsaccades refresh retinal images by moving the receptive fields of less adapted photoreceptors over stationary stimuli, thereby preventing perceptual fading (Ditchburn and Ginsborg, 1952; Martinez-Conde, 2006). However, the locus and properties of this retinal adaptation are not well known.

Therefore, the mechanism of microsaccades for counteracting visual fading is not well understood. This is largely because the neural correlates responsible for brain responses to microsaccades are unknown. So far, the brain responses due to microsaccades have been widely reported at different levels—from neuronal activities (Bair and O'Keefe, 1998; Leopold and Logothetis, 1998; Martinez-Conde et al., 2002; Martinez-Conde, 2006) to electroencephalogram (EEG) (Yuval-Greenberg et al., 2008; Dimigen et al., 2009) and functional magnetic resonance imaging (fMRI) (Hsieh and Tse, 2009; Tse et al., 2010)—in a number of brain areas. It's mostly found that, microsaccades enhance neuronal firing and therefore raise excitatory response in early visual areas, such as lateral geniculate nucleus (LGN) and primary visual cortex (V1) (Martinez-Conde et al., 2002). Particularly, neural activity in V1 is crucial component for the understanding of visual information processing related to microsaccades.

Previous works (Riggs et al., 1953; Krauskopf, 1957; Sharpe, 1972; Engbert and Mergenthaler, 2006) have suggested that retinal adaptation might be responsible for visual fading in the absence of microsaccades during fixation. However, this suggestion has not yet been verified directly in experiments. Moreover, the retinal adaptation in the absence of microsaccades has not been successfully described by using physiologically realistic model (Donner and Hemilä, 2007). Although some studies have found that microsaccades can increase the neural activity in the retina (Armington and Bloom, 1974; Greschner et al., 2002), the enhanced neural responses, which are the neural correlates of the perception of visibility during fading, have been only tested in LGN and V1 but not in the retina (Martinez-Conde et al., 2002; Martinez-Conde, 2006). While retinal adaptation cannot be excluded to contribute to visual fading in the absence of microsaccades, it is possible that the neural adaptation related to visual fading may take place at some stage between retina and early visual areas.

Over the past three decades, physiological studies have shown adaptation phenomena affecting neural activity in V1. Carandini et al. (2002) suggested three possible adaptation mechanisms: synaptic depression, intracortical inhibition and intrinsic cellular mechanisms. Of these three mechanisms, synaptic depression is well suited to explain the marked differences between the responses to transient and consecutive stimuli (Chance et al., 1998). Recently, a synaptic depression, short-term depression (STD), has been extensively found at thalamocortical synapses from LGN to V1 *in vitro* (Stratford et al., 1996; Bannister et al., 2002) and *in vivo* (Boudreau and Ferster, 2005) in the cat. Previously, network models of V1 neurons with the thalamocortical synaptic depression have been used to successfully explain some visual phenomena (Chance et al., 1998; Chance and Abbott, 2001; Carandini et al., 2002), including temporal phase shifts, spatial-phase adaptation, contrast saturation, cross-orientation suppression, and so on. However, the synaptic depression has not yet been used in a thalamocortical network to investigate the roles of microsaccades.

In this paper, we proposed an alternative explanation for visual fading by introducing STD in the thalamocortical system, without considering possible neural adaptation from retina. We used a computational model to investigate how microsaccades might induce neural responses in V1 by considering STD in thalamocortical synapses from LGN to V1. The adapted synapses subjected to STD can lead to response depression in V1, and induce visual fading because of sustained depression. Therefore, it is possible that the generation of microsaccades serves to counteract STD-induced depression of neuronal activity in order to counteract visual fading. Our model can reproduce several experimental findings of microsaccade-related neural responses (Martinez-Conde et al., 2002; Kagan et al., 2008). These results suggest that STD from LGN to V1 might play an important role in microsaccade-related neural responses, and provide theoretical insight into the understanding of more behavioral properties and functional roles of microsaccades.

## 2. Materials and methods

### 2.1. Feedforward model

**Figure 1:**
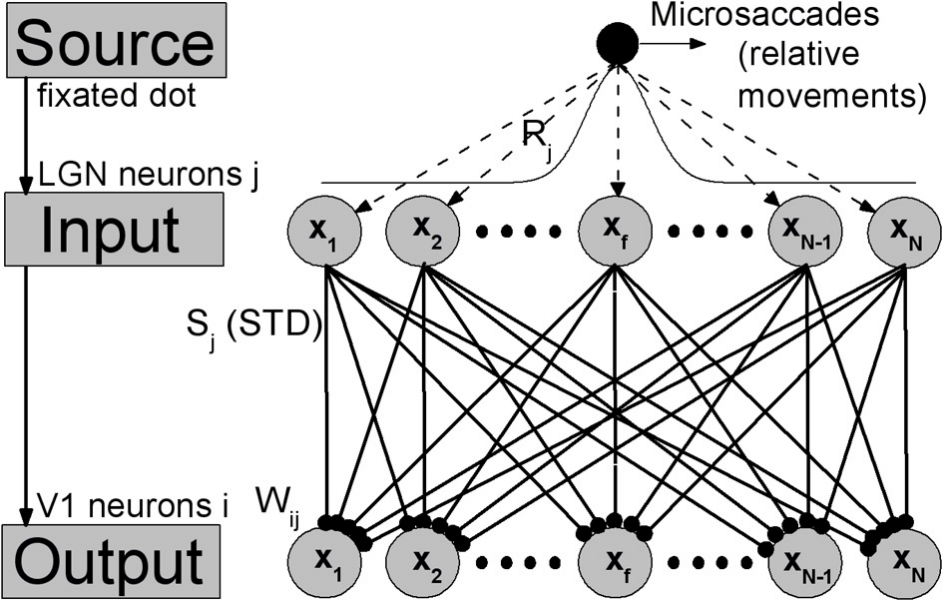
**The feedforward network model during fixation with microsaccades.** Neurons in LGN and V1 are labeled and arranged by the center positions *x*_*j*_ and *x*_*i*_ of their receptive fields in the ranges from −*L* to *L*, respectively. Gaussian filters (receptive fields) in LGN layer transform the afferent stimuli evoked by fixated dot into the inputs with Gaussian firing rate profile: *R*_*j*_ = *G*_1_(*x*_*j*_ − *x*_*f*_) = *A* exp^−(*x*_*j*_ − *x*_*f*_)^2^/σ^2^_1_^. *A* represents the amplitude of a visual input at fixated-dot position *x*_*f*_ and σ_1_ is the width of the tuning curve, which denotes the width of the receptive fields. The output layer V1 is connected to input layer LGN by thalamocortical synapses with synaptic strengths *S*_*j*_, which are subjected to the synaptic modification: STD. These connecting weights *W*_*ij*_ follow the Gaussian tuning curve: *W*_*ij*_ = *G*_2_(*x*_*j*_ − *x*_*i*_) = exp^−(*x*_*j*_ − *x*_*i*_)^2^/σ^2^_2_^, where *x*_*j*_-*x*_*i*_ denotes the position difference of receptive field centers between the input neuron *j* and the output neuron *i*. The microsaccades during fixation can be regarded as instantaneous relative movements of the fixated dot over LGN with microsaccadic magnitude Δ_*M*_. In order to eliminate the effect of boundary due to limited network scale, the *G*_1_ is extended to a period boundary function, i.e., *G*_1_(*x*_*j*_ − *x*_*f*_) = *A* exp^−(*x*_*j*_ − *x*_*f*_)^2^/σ^2^_1_^ for |*x*_*j*_ − *x*_*f*_| < *L*; otherwise, *G*_1_(*x*_*j*_ − *x*_*f*_) = *A* exp^−(2*L* −|*x*_*j*_ − *x*_*f*_|)^2^/σ^2^_1_^.

In sensory nervous system, substantial information processing can be performed by feedforward networks without considering recurrent connections, including perceptual learning (Tsodyks and Gilbert, 2004). A well-known model of feedforward networks is that proposed by Poggio et al. (1992) on visual hyperacuity. In our work, extending this previous model, we constructed a simple feedforward network model consisting of two layers corresponding to LGN and V1 with STD in thalamocortical synapses, as shown in Figure 1. To focus on the effects of synaptic depression during fixation with microsaccades, we kept our model very simple, and did not consider corticocortical synaptic connections. In visual systems, a neuron sees only a small portion of the visual field. This small area is called the receptive field of the cell. This receptive field leads to a Gaussian tuning function of the mean firing rate of the neurons with respect to the orientation of fixated dot (Nelson et al., 1994; Ferster et al., 1996; Ferster and Miller, 2000; Seriès et al., 2004), which denotes the inputting orientation from fixated dot to the neurons by lateral synaptic inhibition connections (Amari, 1977; Pinault and Deschênes, 1998; Yuan et al., 2006, 2007). Since the input layer LGN consists of a number of Gaussian filters (receptive fields) as described by Poggio et al. (1992) and Tsodyks and Gilbert (2004), the afferent stimuli evoked by the fixated dot are transformed into firing trains in LGN neurons *j*, which can be described as Poisson spike trains with a time-independent rate *R*_*j*_ following Gaussian profile *G*_1_ in space (shown in Figure 1). For the output layer, each V1 neuron *i* has connections coming from LGN relay neurons *j* (excitatory) with weights *W*_*ij*_ following Gaussian tuning curve *G*_2_ (explained in the caption of Figure 1) (Poggio et al., 1992; Tsodyks and Gilbert, 2004). The model is composed of Integrate-and-Fire neurons with chemical couplings of δ function. The dynamics of the membrane potential *V*_*i*_ of output neuron *i* in V1 is described by

(1) $$\tau_{m}\frac{dV_{i}}{dt}=V_{0}-V_{i}+\sum_{j=1}^{N}gW_{ij}S_{j}(t)(V_{E}-V_{i})\delta (t-t_{sp}^{j}).$$

Here, we adopted the same parameter values as those in Abbott et al. (1997) and Chance et al. (1998), which model V1 cells according to empirical observations (Varela et al., 1997). The membrane time constant τ_*m*_ equals 30 ms, the resting potential *V*_0_ is −70 mV, and the reversal potentials *V*_*E*_ for all the excitatory synapses are 0 mV. Each V1 neuron *i* integrates inputs coming from LGN neurons *j* at spike time *t*^*j*^_*sp*_ distributed as Poisson spike trains. When the potential *V*_*i*_ reaches the threshold value −55 mV, the neuron *i* emits a spike, and then the membrane potential is reset to the relatively high value −58 mV (compared with the resting potential *V*_0_ = −70 mV) in order to match experimental recordings (Varela et al., 1997). The parameter *g* represents the maximal synaptic conductance. The *S*_*j*_(*t*) in the thalamocortical synapses from LGN to V1 complies with STD plasticity, which will be described in the following.

In simulations, *N* neurons in LGN and V1 are, respectively, spread uniformly in the ranges from −*L* to *L*, which denote the physical positions of receptive field centers of these neurons. Compared with the responsive region of neurons induced by the fixated dot, *L* should be large enough that the new place of fixated dot after microsaccades is far from the boundary neurons. Here, in order to shorten the simulating time, the region from −*L* to *L* is chosen as a narrow region with finite *L*. Meanwhile, to eliminate the effect of boundary due to the chosen narrow region, the input tuning curve *G*_1_ is extended to a period boundary function (see the caption of Figure 1). In this way, the value of *L* does not change qualitatively the results. In our simulation, microsaccades are modeled by instantaneous relative displacement Δ_*M*_ of the tuning curve *G*_1_. With suitable scale transformation, the size *L* and displacement Δ_*M*_ can be used to represent realistic range of microsaccades (Martinez-Conde et al., 2009). Here, we take *N* = 1000 neurons and *L* = 10. The main results, however, do not depend on these parameters.

### 2.2. Short-term depression (STD)

Biophysically, synaptic depression can be regarded as the interaction between two processes, the activity-dependent depletion of the transmitter resources of synaptic vesicles and the slow replenishment of the resources. The depletion process means that the available transmitter is diminished immediately after the presynaptic spike time owing to the release of transmitter. Thus, each time a presynaptic spike arrives at synapse *j*, the synaptic strength *S*_*j*_ decreases immediately after the spike due to the use of transmitter resources. The depletion of a synapse is usually modeled by a multiplicative factor *f* (Abbott et al., 1997; Chance et al., 1998; Boudreau and Ferster, 2005):

(2) $$S_{j}\to fS_{j}.$$

The parameter *f* (0.0 < *f* < 1.0) denotes the ratio of the synaptic resources available immediately after release to those before release, and thereby determines the amount of depression at synapse *j* induced by each spike (the smaller the parameter *f*, the stronger the depression). The slow replenishment process can be modeled by exponential recovery from depression (Abbott et al., 1997; Chance et al., 1998):

(3) $$\frac{dS_{j}}{dt}=\frac{1}{\tau_{S}}(1-S_{j}).$$

The constant parameter τ_*S*_ determines the depression recovery time. Combining the Equations (2) and (3), the STD can be described by

(4) $$\frac{dS_{j}}{dt}=\frac{1}{\tau_{S}}(1-S_{j})-(1-f)S_{j}\delta (t-t_{sp}^{j}).$$

If the afferent neuron for the synapse *j* fires a Poisson spike train at rate *R*_*j*_, the synaptic strength will quickly decrease to the approximate steady state (for a high rate) (Abbott et al., 1997):

(5) $$S_{j}(ss)=\frac{1}{f+(1-f)R_{j}\tau_{S}},$$

when the depletion and replenishment processes reach a balance. According to the property of synaptic depression, it is obvious that the microsaccade can increase the activity in the nearby V1 neurons that have the receptive field of the landing position in our proposed feedforward network. This synaptic depression model gives a good fit of experimental data (Abbott et al., 1997). The two parameter values *f* and τ_*S*_ we used lie within the ranges indicated in the experimental data (Carandini et al., 2002; Boudreau and Ferster, 2005). In the following computations, we took *f* = 0.75 and τ_*S*_ = 200 ms. Choosing different parameter values does not alter the qualitative results.

## 3. Results

By using our feedforward model, we first describe the microsaccade-induced excitatory activity in V1 neurons that might contribute to counteract perceptual fading. Then, we will show that our model can reproduce experimental observations about V1 cortical responses after microsaccades as reported by Martinez-Conde et al. (2000, 2002). Moreover, our model can explain the saturation property of visual brain responses for large microsaccadic magnitude and velocity, which has been recently found by measuring scalp EEG (Dimigen et al., 2009).

Since microsaccades are very fast movements (Martinez-Conde et al., 2009), for simplicity, we ignored the time course of microsaccades (Donner and Hemilä, 2007) in most of our simulations, i.e., the displacement by microsaccade of magnitude Δ_*M*_ happens immediately. However, we also studied the impact of velocity and showed that it also reproduces experimental findings. Here, we counted the total number of spikes *N*_*sp*_ of the V1 neurons in the model in a moving time bin (*T* = 50 ms) as a measure of the neural response.

### 3.1. Excitatory responses to microsaccades and a possible explanation for counteracting visual fading

**Figure 2:**
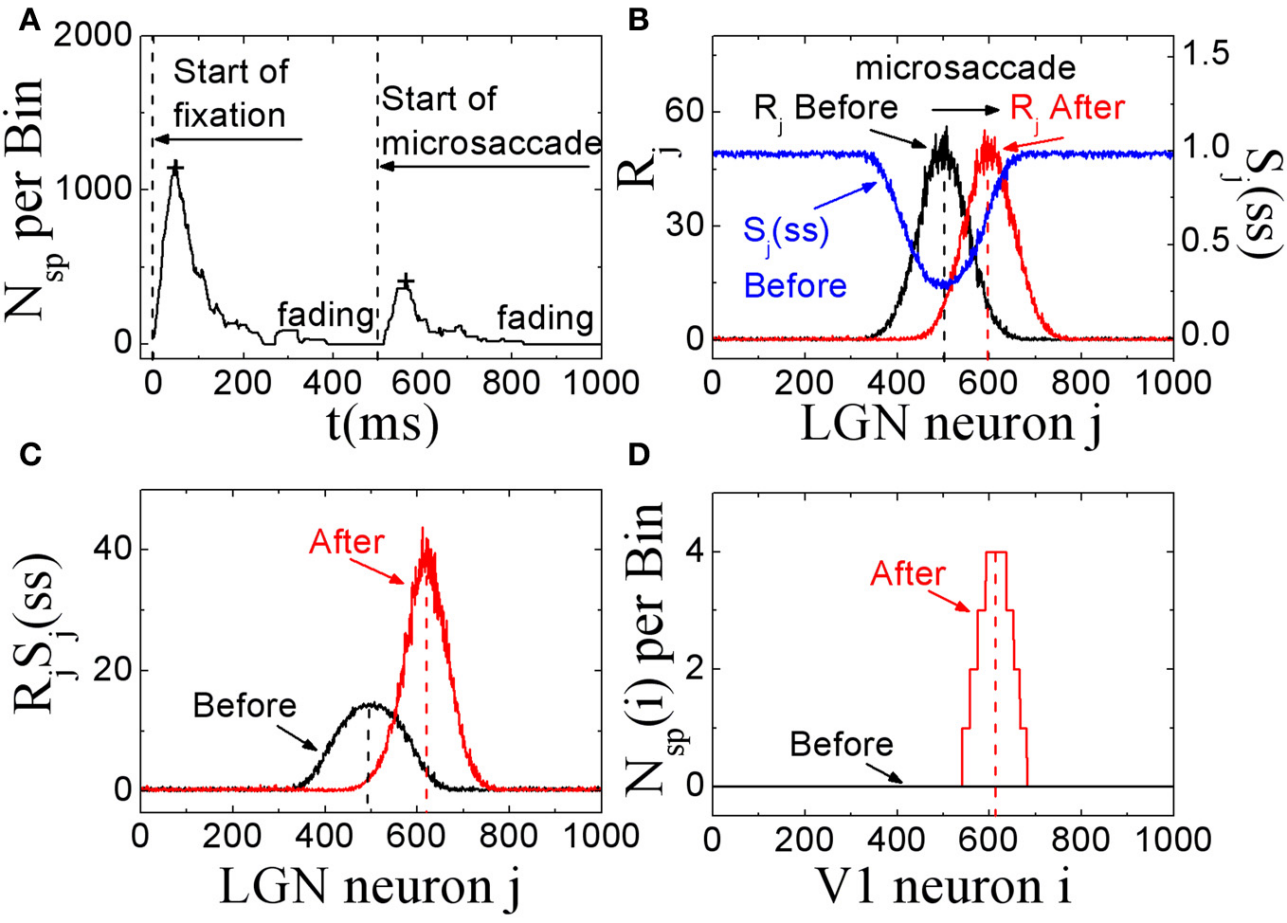
**The excitatory responses to microsaccades during fixation in the feedforward network model. (A)** The change of neuronal activity in V1 neurons before and after microsaccade. The signs “+” reflect the response peaks. **(B)** The computer-generated input firing rates *R*_*j*_ in LGN neurons before and after microsaccade, and the STD-modified steady states *S*_*j*_(*ss*) of synaptic strengthens before microsaccade. **(C)** The synaptic input *R*_*j*_*S*_*j*_(*ss*) for each LGN neuron *j* before and after microsaccade. **(D)** The neuronal spike number *N*_*sp*_(*i*) per time bin (50 ms) of the output neuron *i* in V1 before and after microsaccade. Here the parameters are *g* = 0.15, *A* = 50, σ_1_ = σ_2_ = 1.5, and Δ_*M*_ = 2.0.

In these simulations, we showed that, STD in thalamocortical synapses can provide a possible explanation for microsaccades in counteracting visual fading. Here, we assumed that the fixation dot is the only relevant visual stimulus that generates the visual signal. As shown in Figure 2A, in this model with STD, neural activity in V1 begins to fade within several hundred milliseconds after the start of fixation in the absence of fixational eye movements (and head or body movements). If a microsaccade occurs, the neural excitation will return and persist for a few hundred milliseconds. If there are no more microsaccades, the neural activity in V1 will be fading completely in about 300 ms. In Figures 2B–D, we propose an explanation of the responses in terms of STD in thalamocortical synapses. During fixation in the absence of microsaccades, the spike trains evoked by the fixated dot with firing rates *R*_*j*_ in LGN persist in stimulating thalamocortical synapses (Figure 2B, black line). Due to depressing mechanism of STD in these synapses, the synaptic strengths will quickly decrease to steady state values. The strengths *S*_*j*_ with larger firing rate *R*_*j*_ will decrease to smaller steady state values *S*_*j*_(*ss*) (Figure 2B, blue line). The theoretical analysis (Abbott et al., 1997) showed that the steady state strengths *S*_*j*_(*ss*) are inversely proportional to *R*_*j*_ for high firing rates (see Equation 5). When there is a microsaccade, the network will generate a new neural input to stimulate V1 neurons by moving the fixated dot over the receptive fields of LGN neurons with less adapted thalamocortical synapses (in the sense of relative movement; Figure 2B, red line). Before the microsaccade, the input of each thalamocortical synapse from LGN neuron *j*, which is proportional to *R*_*j*_*S*_*j*_(*ss*) (Abbott et al., 1997), is rather small (Figure 2C, black line), and does not induce firing of V1 neurons for the parameter we used (Figure 2D, black line). However, immediately after the microsaccade, the new input of each thalamocortical synapse with less adaptation becomes much larger (Figure 2C, red line) due to the fast eye movement so that it can evoke spikes in V1 neurons (Figure 2D, red line). Afterwards, STD becomes effective to reduce the synaptic strengths and the response fades out again. These simulations indicate that, STD in thalamocortical synapses can give a potentially valid explanation for microsaccades in countering visual fading, which may suggest an important role of STD in microsaccade-related neural responses during fixation.

**Figure 3:**
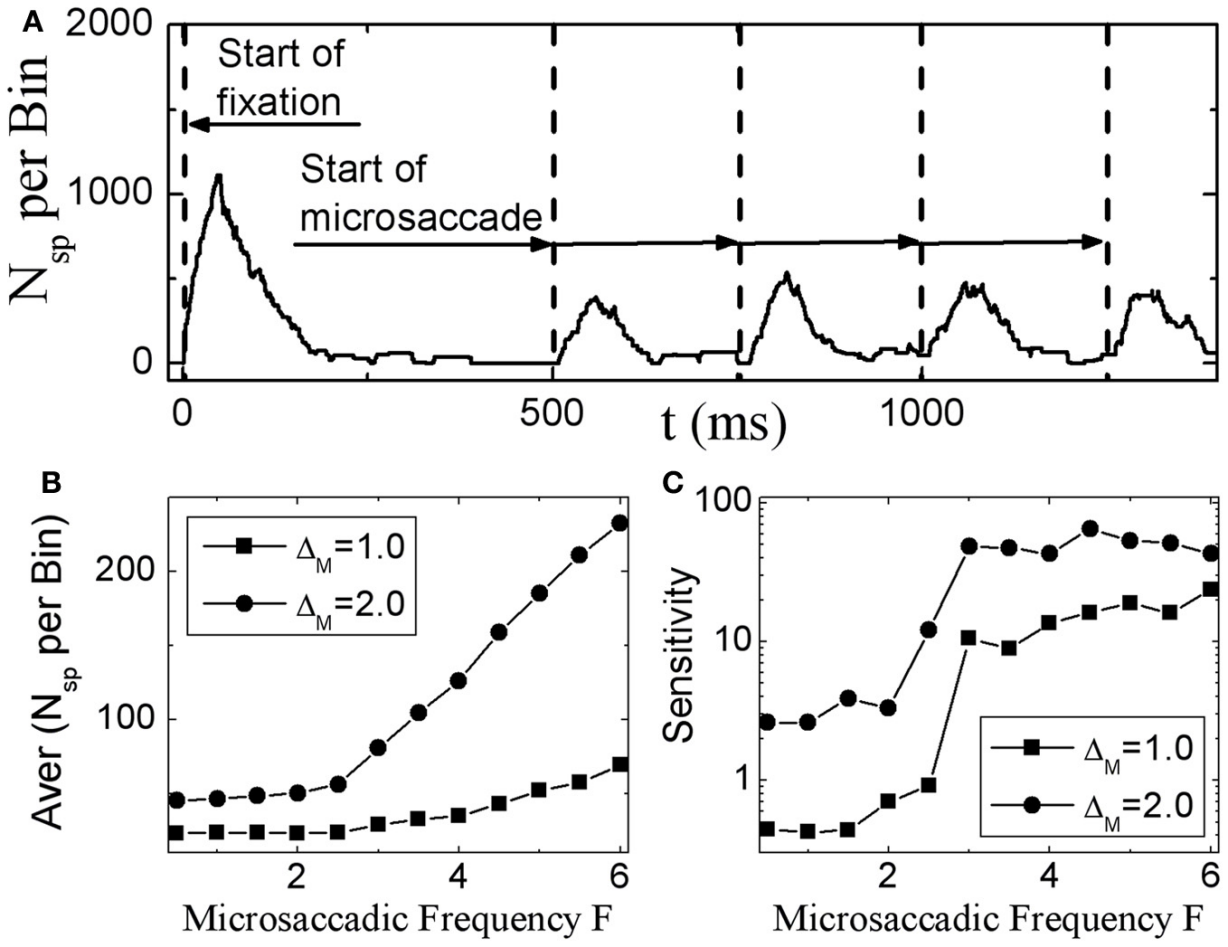
**Sustained responses in V1 neurons to microsaccades with high enough frequency. (A)** The sustaining neuronal activity induced by periodic microsaccades with a frequency *F* = 4 Hz. The average neuronal activity during fixation **(B)**, and its responsive sensitivity **(C)** correlated with random microsaccades (in Poisson trains) as a function of microsaccadic frequency *F* for different microsaccadic sizes Δ_*M*_. Here the parameters are *g* = 0.15, *A* = 50, and σ_1_ = σ_2_ = 1.5. In **(A)**, Δ_*M*_ = 2.0 is chosen.

Next, we investigate in more detail the effect of microsaccadic frequency. As shown in Figure 3A, neural activity in V1 is sustained and does not fade away if there are microsaccades with high enough frequency. Here, we calculate the average neural activity related to microsaccades during fixation as a function of microsaccadic frequency *F* (Figure 3B). It is found that, the neural activity will start to increase obviously when microsaccadic frequency increases to 3–4 Hz. We also quantify the sensitivity of neuronal response to change of frequency *F* by an amount Δ*F*, which is the slope of average neural activity curve as the function of *F* in Figure 3B. As shown in Figure 3C, the sensitivity increases to high enough value when microsaccadic frequency arrives to 3–4 Hz. These results indicate that, the neural activity will be sustained and sensitive if the frequency of microsaccades or macrosaccades is about 3–4 Hz, which is consistent with the fact that microsaccades occur 3–4 times per second (Otero-Millan et al., 2008; Martinez-Conde et al., 2009), though the real situation could be more complicated to involve other factors such as the variable sizes and speeds of the microsaccades.

### 3.2. Reproducing experimental observations

#### 3.2.1. Different responses to microsaccades with flashing and stationary stimuli

**Figure 4:**
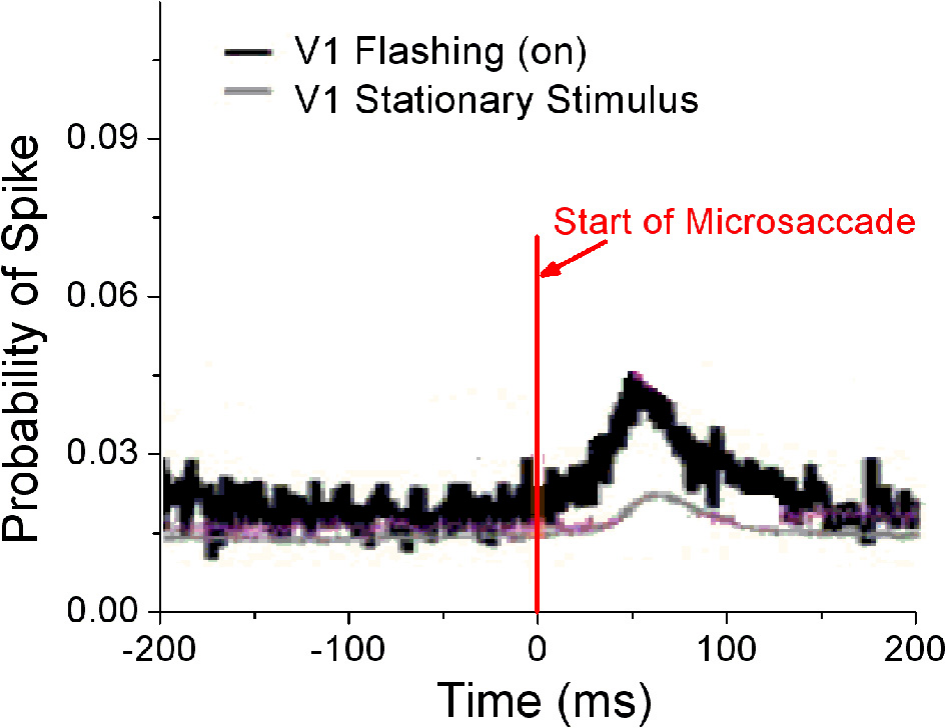
**Experimental data for probability of neural spikes in V1 when the fixated dot is stationary or flashing (on).** [Adapted from Martinez-Conde et al. (2002)].

**Figure 5:**
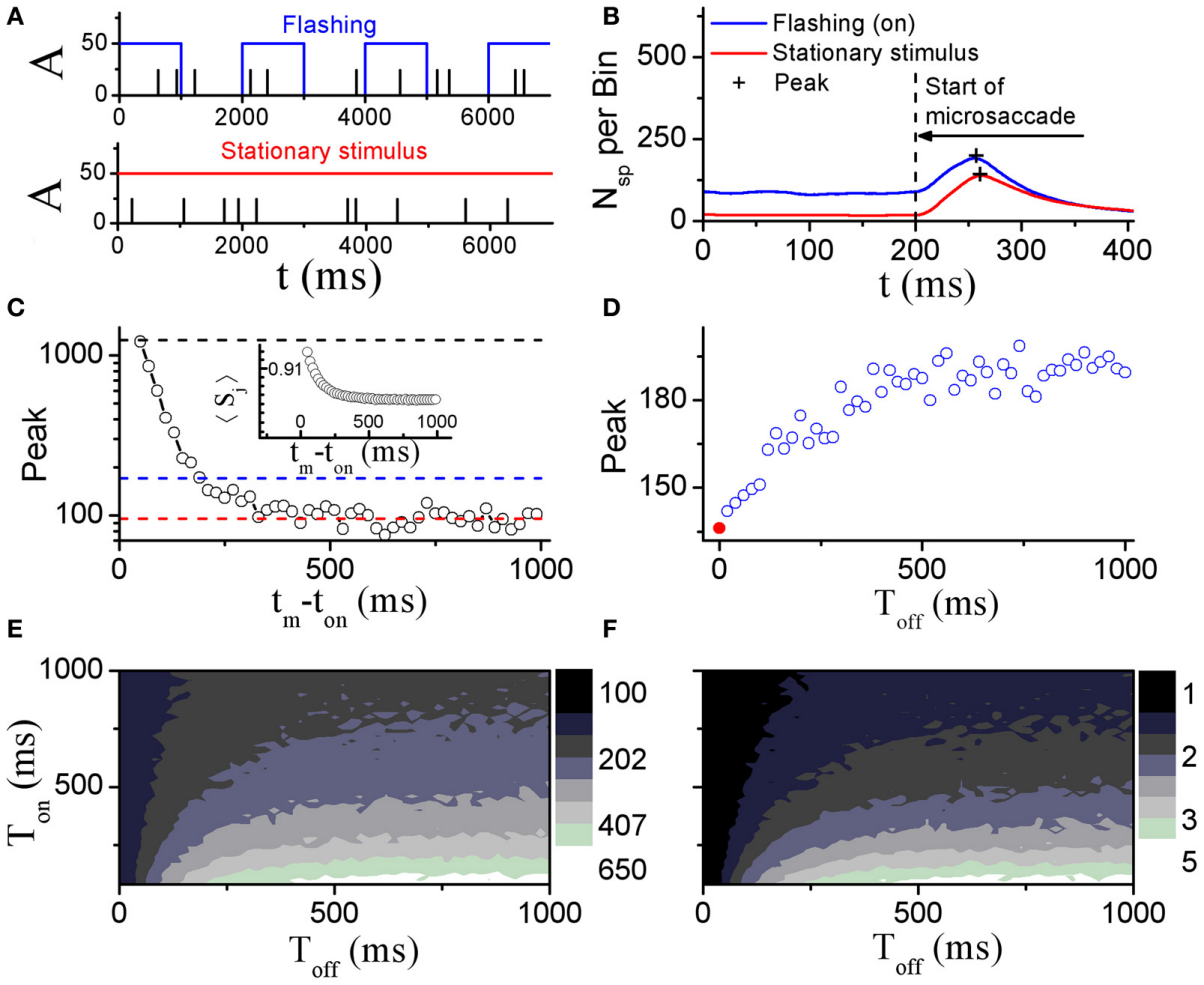
**Comparison of model neural responses to microsaccades in V1 when the fixated dot is stationary or flashing cyclically with *T*_*on*_ and *T*_*off*_ as indicated by the blue rectangles. (A)** Stimulus brightness *A* (see Figure 1) of the fixated dot for a periodic flashing condition (blue; *T*_*on*_ = 1 s, *T*_*off*_ = 1 s) and stationary presentation (red; constant *A*) in the presence of microsaccades (black ticks, with fixed size Δ_*M*_) in Poisson trains (1.5 Hz, here we choose the smaller frequency of microsaccades than the real microsaccadic frequency in order to avoid the correlated neural activity from one microsaccade to another due to the Poisson microsaccade trains in simulation). **(B)** Microsaccade increases neural activity in V1 when the fixated dot is stationary, and further increases the neural activity when it is flashing-on. “+”-signs denote the peaks of microsaccade-related neural activities. The results are obtained by averaging over all microsaccades in Poisson trains (1.5 Hz) during the on-state. **(C)** The response peak (i.e., the second response peak in Figure 2A) evoked by a microsaccade as a function of the interval *t*_*m*_ − *t*_*on*_ between onset of microsaccade and onset of flashing-on state. The mean values over all *t*_*m*_ − *t*_*on*_ from 0 to *T*_*on*_ (blue dashed line) and the baseline of peak response (red dashed line) are approximately equal to the response peaks “+” in **(B)**. In addition, the response peak (the first response peak in Figure 2A) induced by onset of stimulus is plotted (black dashed line). Inset in **(C)**: network-averaged synaptic strength 〈*S*_*j*_〉 as a function of *t*_*m*_ − *t*_*on*_. **(D)** Response peaks “+” in **(B)** as a function of the off duration *T*_*off*_ of the flashing dot. The red • at *T*_*off*_ = 0 corresponds to the stationary stimulus. **(E)** Phase diagram in *T*_*off*_ − *T*_*on*_ plane for the response peak “+” in the flashing condition in **(B)**. **(F)** As in **(E)**, but for the ratio of the two response peaks in **(B)** (flashing (on) to stationary stimulus). Here, data are obtained from simulation for 1000 s in **(B)**, **(D–F)** and averaged over 20 realizations for **(C)**. The other parameters are *g* = 0.15, σ_1_ = σ_2_ = 1.5, and Δ_*M*_ = 1.0.

Perceptual responses to flashed object have been experimentally studied in the presence of microsaccades (Martinez-Conde et al., 2002; Kagan et al., 2008) and saccade (Lappe et al., 2006). Particularly, Martinez-Conde et al. (2002) has experimentally compared neural activities in V1 induced by microsaccades with flashing and stationary (non-flashing) stimulus bars, in order to study how effective microsaccades are in generating neural activity by comparing them with previously characterized and well-known visual stimuli, flashing bars. In their experiment, the stimulus bars were in the receptive fields of the recorded V1 neuron both before and after the microsaccade. They used a white bar on a black background for on cells, and a black bar on a white background for off cells. Then, they calculated the spike probability of neurons in V1 to reflect neural response. As shown in Figure 4, the neural response after microsaccades is stronger when a rhythmically flashing bar is on during fixation, as compared to a condition in which the stimulus bar is always on (stationary). Here, our model can provide a possible understanding of this observation using STD. We considered that the fixated dot as stimulus can be flashing (on–off) or stationary (Figure 5A). As shown in Figure 5B, the baseline before microsaccades and responsive peak after microsaccades when the flashing dot is on are both higher than those when the fixated dot is stationary, consistent with the experimental findings by Martinez-Conde et al. (2002). The higher responses are expected because of the additional onset response when the flashing bar is turned on. Namely, in our model, the observations are expected to be due to smaller synaptic depression during the shorter interval between the onset of the flashing-on and the onset of a microsaccade. To further understand the mechanism, we examined how the neural response Figure 5C) and network-averaged synaptic strength 〈*S*(*j*)〉 (inset of Figure 5C) depend on the time interval *t*_*m*_ − *t*_*on*_ between the onsets of microsaccade and flashing-on. A smaller interval corresponds to a higher response activity due to a larger synaptic strength. When the interval *t*_*m*_ − *t*_*on*_ is larger, the neural response will decrease to a relatively stable baseline (Figure 5C, red dashed line) due to the presence of a large final stable synaptic depression after the larger interval (Figure 5C, inset). This baseline is the approximate response with the stationary stimulus since the synaptic strength for this case decreases to the same stable value as that for stationary stimuli. Because the time interval between the onsets of microsaccade and flashing-on is random (Martinez-Conde et al., 2002), the average response to all the microsaccades during flashing-on is approximately equal to the average activity over the whole possible intervals (i.e., 0 ≤ *t*_*m*_ − *t*_*on*_ ≤ *T*_*on*_, where *T*_*on*_ is the duration of flashing-on; Figure 5C, blue dashed line). Clearly, the interval-averaged response (Figure 5C, blue dashed line) is larger than the baseline, explaining that the microsaccade-related response with flashing (on) stimulus is higher than that with stationary stimulus. Moreover, we compared the microsaccade-related responses to the response after a flashing bar turns on. The response after a flashing bar turns on (Figure 5C, black dashed line) is several times larger than the two microsaccade-related responses with flashing (Figure 5C, blue dashed line) and stationary stimuli (Figure 5C, red dashed line), consistent with the experimental observations in Martinez-Conde et al. (2002) and Kagan et al. (2008). Obviously, this is because the synaptic strengths with the thorough recovery from STD within *T*_*off*_ = 1 s are involved in the neural response after a flashing bar turns on, which is the same as the response at the start of fixation (the first response peak in Figure 2A).

To further study microsaccade-related neural responses due to STD with a flashing stimulus, we investigated effects of the flash-on duration *T*_*on*_ and the flash-off duration *T*_*off*_ (the time that passed since the last flash onset or offset, respectively) (Figures 5D–F). During the off state, the synaptic strengths will recover from the depression, reaching larger synaptic strengths with longer *T*_*off*_ till saturation. Thus, the microsaccade-related neural response increases with increasing *T*_*off*_ and then reaches saturation due to the thorough synaptic recovery for the larger *T*_*off*_ (Figure 5D). For the effect of the on-duration *T*_*on*_, we can infer from Figure 5C that the neural response (Figure 5C, blue dashed line) will become larger with the decrease of *T*_*on*_. Therefore, the ranges of the observed increase of microsaccade-related neural response and of the increased ratio of the response with flashing stimuli relative to stationary stimuli are in line with large *T*_*off*_ and small *T*_*on*_ (Figures 5E and F).

#### 3.2.2. Saturation of activity for large microsaccadic magnitude and velocity

**Figure 6:**
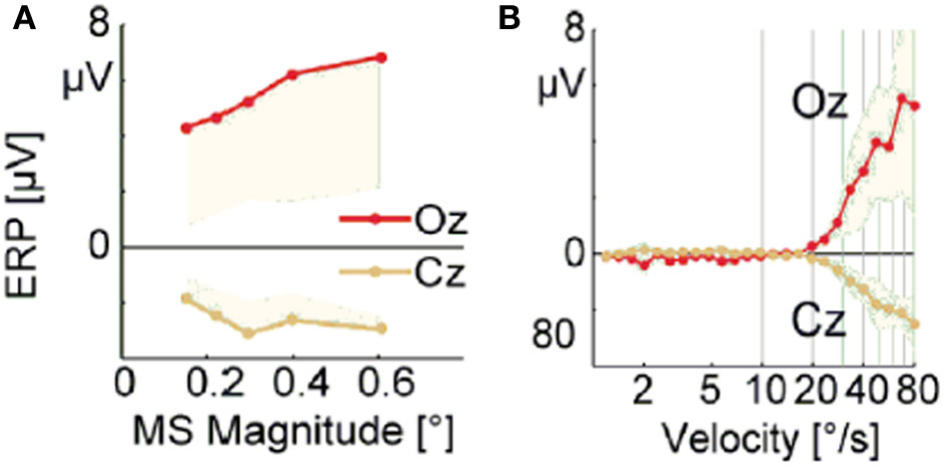
**Experimental data of EEG voltage at the occipital electrode Oz (red) and the vertex Cz (orange) as a function of microsaccade magnitude (A) and eye movement velocity (B).** [Adapted from Dimigen et al. (2009)]

**Figure 7:**
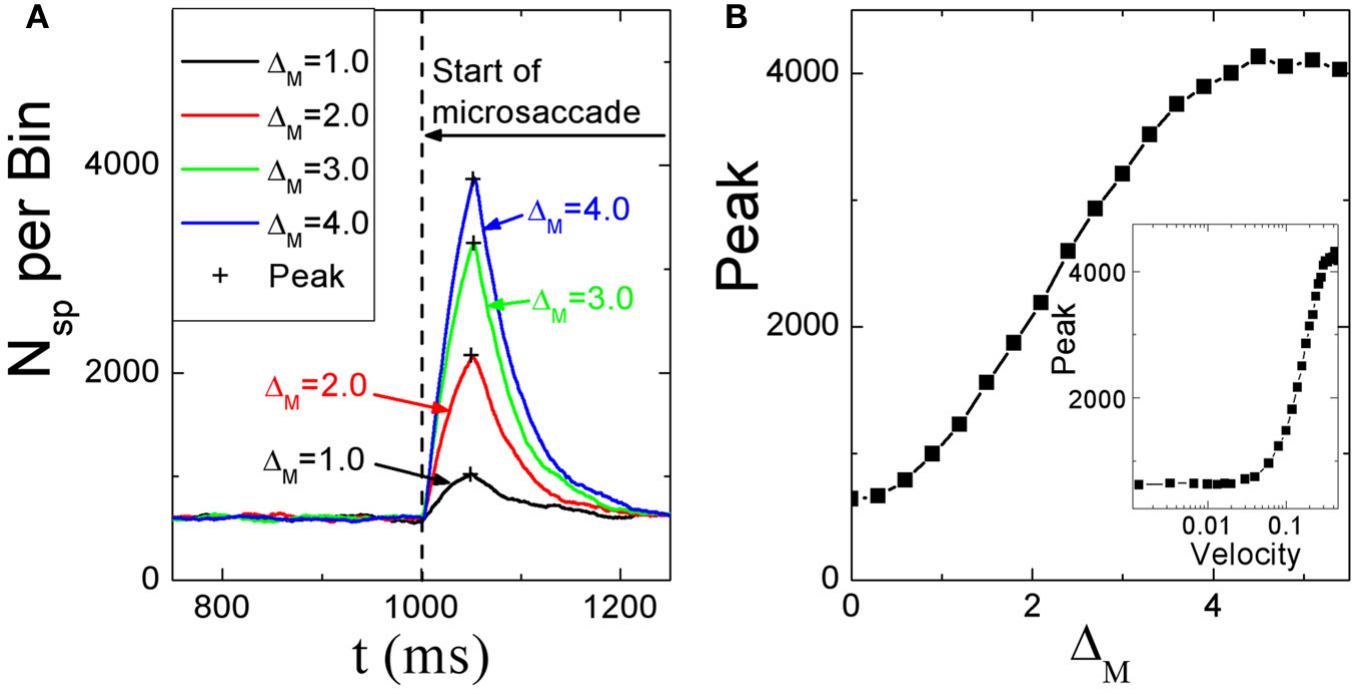
**(A)** Microsaccade-induced neural activity for different microsaccade magnitudes. After microsaccade, there is a response peak. **(B)** Saturation of the response peak for large microsaccades. The inset in **(B)**: the effect of microsaccadic velocity on response peak in Log-Linear scale for comparison with Figure 6B, experimentally found in Dimigen et al. (2009). Here *g* = 0.2, *A* = 100, and σ_1_ = σ_2_ = 1.5. Data are averaged over 20 independent runs.

Dimigen et al. (2009) studied microsaccade-related brain activity in event-related brain potentials (ERP). ERP is the average of many epochs of EEG trials recorded from scalp for the same task, synchronized to the same event such as the stimulus onset or microsaccade onsets, yielding a clear pattern of brain response to the external signal when compared to the base line. (Picton et al., 2000; Handy, 2005; Ouyang et al., 2011). Dimigen et al. (2009) found that the tiny eye movements by microsaccades can generate sizable visual brain response in ERP comparable to usual saccadic eye movements and responses correlated with microsaccades tend to saturate for large microsaccades (Figure 6A, red line). Our model can provide a possible explanation for this phenomenon by the effect of microsaccade magnitude on neural activity, shown in Figure 7. A response peak appears soon after the microsaccade, and the value increases with the microsaccade magnitude Δ_*M*_ (Figure 7A). The increase is almost linear for small microsaccades, consistent with the finding by Dimigen et al. (2009). As the microsaccade magnitude increases further, the increasing response reaches saturation (Figure 7B). This saturation can be explained as follows. As shown in Figures 7B and C, the synaptic input *R*_*j*_*S*_*j*_ increases after a microsaccade by moving the fixated dot over the receptive fields of LGN neurons with less adapted thalamocortical synapses (in the sense of relative movement). But, when the moving distance due to large microsaccade exceeds the region with strong synapse-depression, the synaptic input will become independent of the microsaccade magnitude, leading to saturated response.

So far, all the above simulations were done without considering the finite velocity of microsaccade. In Figure 6B, a relationship between instantaneous eye movement velocity (also including periods of drift) and the amplitude of occipital EEG response 100 ms later was observed by Dimigen et al. (2009). Our model can provide an understanding of this experimentally observed relationship when microsaccadic velocity is taken into consideration. In the simulation, we assume a constant velocity for microsaccadic movements, with a fixed duration of 15 ms for microsaccades of different sizes, following the experimental findings that there are approximately fixed microsaccadic durations (around 15 ms for human) for different microsaccadic velocities (Troncoso et al., 2008; Dimigen et al., 2009). The results shown in the inset of Figure 7B agree well with the pattern shown in Figure 6B, experimentally found in Dimigen et al. (2009). Though the analysis in Dimigen et al. (2009) included all samples of the eye movement trajectory, not only microsaccades, it is reasonable to believe that most of the medium-velocity samples belong to microsaccades (Martinez-Conde et al., 2004, 2009). From our model, we can understand that, at slow eye velocities, retinal displacements are small and the signal moves only slightly and slowly away from the strongly depressed region, without inducing strong neural response. This may be able to explain that tremor and drift do not induce significant neural responses (Gur et al., 1997; Martinez-Conde, 2006). With larger velocities, the signal quickly moves to a much less depressed region and induces sizable response.

## 4. Discussion and conclusion

The prevailing theory suggests that visual fading in the absence of microsaccades is caused by retinal adaptation. However, retinal adaptation for visual fading has not been directly tested in experiments. On the other hand, STD in thalamocortical systems from LGN and V1 has been empirically confirmed and could play an important functional role. Based on these considerations, we have proposed an alternative potential biophysical foundation for the explanation how microsaccades counteract visual fading, thalamocortical STD. With a simple feedforward model, we showed that, without considering possible retinal adaptation, STD from LGN to V1 alone can qualitatively reproduce several experimental observations about microsaccade-induced brain responses.

However, it is important to note that these two possible mechanisms of retinal adaptation and STD are not mutually exclusive. In fact, enhanced LGN activity by microsaccades as observed in experiments Martinez-Conde et al. (2000, 2002) could be an indication of possible retinal adaptation. In the real visual systems, the two possible mechanisms could yield different functional benefits for visual information processing, which are yet unknown. If retinal adaptation could be effectively described similar to STD and retinal neurons can be described similarly as in LGN and V1, and assuming that there is no STD in thalamocortical synapses, then from the viewpoint of simplified feedforward neural model, the response in V1 could be similar to what we described here for STD from LGN to V1. Possibly different functional/behavioral effects of the two mechanisms then would rely strongly on the biophysical details. Perhaps the most interesting possibility is that these two adaptation levels are actually arranged in a cascade. Such a cascading of adaptation is expected to enhance the sensitivity of adaptation, likely to sharpen the cortical neural responses to tiny and fast eye movements (or equivalently tiny and fast movement of the visual world). Further investigations are expected to reveal more behavioral properties and functional roles of microsaccades (for review, see Rolfs, 2009). The work presented in this paper will serve as a foundation for future studies.

To sum up, we proposed an alternative synaptic explanation for microsaccades in counteracting visual fading during fixation by introducing STD in the thalamocortical system. Moreover, the depression model can reproduce several experimental observations of microsaccade-related neural responses in V1. Our model and results are expected to provide quantitative method and theoretical insight into the study of microsaccades. Generally, our model may provide a useful tip for the understanding of visual information adaptation and transmission, and give a starting point for modeling visual process of microsaccades by considering more neurobiological ingredients, such as inhibitory connections within V1 and from LGN, and other types of synaptic plasticity and cascading with possible retinal adaptation.

### Conflict of interest statement

The authors declare that the research was conducted in the absence of any commercial or financial relationships that could be construed as a potential conflict of interest.
